# Diabetes changes expression of genes related to glutamate neurotransmission and transport in the Long-Evans rat retina

**Published:** 2013-07-19

**Authors:** Jennifer C.M. Lau, Roger A. Kroes, Joseph R. Moskal, Robert A. Linsenmeier

**Affiliations:** 1 Department of Chemical and Biological Engineering, Northwestern University, Evanston, IL; 2 Falk Center for Molecular Therapeutics, Department of Biomedical Engineering, Northwestern University, Evanston, IL; 3 Department of Biomedical Engineering, Northwestern University, Evanston, IL; 4 Department of Neurobiology, Northwestern University, Evanston, IL; 5 Department of Ophthalmology, Northwestern University, Chicago, IL

## Abstract

**Purpose:**

This study investigated changes in the transcript levels of genes related to glutamate neurotransmission and transport as diabetes progresses in the Long-Evans rat retina. Transcript levels of vascular endothelial growth factor (VEGF), erythropoietin, and insulin-like growth factor binding protein 3 (IGFBP3) were also measured due to their protective effects on the retinal vasculature and neurons.

**Methods:**

Diabetes was induced in Long-Evans rats with a single intraperitoneal (IP) injection of streptozotocin (STZ; 65 mg/kg) in sodium citrate buffer. Rats with blood glucose >300 mg/dl were deemed diabetic. Age-matched controls received a single IP injection of sodium citrate buffer only. The retinas were dissected at 4 and 12 weeks after induction of diabetes, and mRNA and protein were extracted from the left and right retinas of each rat, respectively. Gene expression was analyzed using quantitative real-time reverse-transcription PCR. Enzyme-linked immunosorbent assay was used to quantify the concentration of VEGF protein in each retina. Statistical significance was determined using 2×2 analysis of variance followed by post-hoc analysis using Fisher’s protected least squares difference.

**Results:**

Transcript levels of two ionotropic glutamate receptor subunits and one glutamate transporter increased after 4 weeks of diabetes. In contrast, 12 weeks of diabetes decreased the transcript levels of several genes, including two glutamate transporters, four out of five N-methyl-D-aspartate (NMDA) receptor subunits, and all five kainate receptor subunits. Diabetes had a greater effect on gene expression of NMDA and kainate receptor subunits than on the α-amino-3-hydroxy-5-methyl-4-isoxazole propionate (AMPA) receptor subunits, for which only GRIA4 significantly decreased after 12 weeks. VEGF protein levels were significantly increased in 4-week diabetic rats compared to age-matched control rats whereas the increase was not significant after 12 weeks. Transcript levels of VEGF and VEGF receptors were unchanged with diabetes. Erythropoietin and IGFBP3 mRNA levels significantly increased at both time points, and IGFBP2 mRNA levels increased after 12 weeks.

**Conclusions:**

Diabetes caused significant changes in the transcriptional expression of genes related to ionotropic glutamate neurotransmission, especially after 12 weeks. Most genes with decreased transcript levels after 12 weeks were expressed by retinal ganglion cells, which include glutamate transporters and ionotropic glutamate receptors. Two genes expressed by retinal ganglion cells but unrelated to glutamate neurotransmission, γ-synuclein (SNCG) and adenosine A1 receptor (ADORA1), also had decreased mRNA expression after 12 weeks. These findings may indicate ganglion cells were lost as diabetes progressed in the retina. Decreased expression of the glutamate transporter SLC1A3 would lead to decreased removal of glutamate from the extracellular space, suggesting that diabetes impairs this function of Müller cells. These findings suggest that ganglion cells were lost due to glutamate excitotoxicity. The changes at 12 weeks occurred without significant changes in retinal VEGF protein or mRNA, although higher VEGF protein levels at 4 weeks may be an early protective response. Increased transcript levels of erythropoietin and IGFBP3 may also be a protective response.

## Introduction

Diabetic retinopathy, a major complication of type 1 and 2 diabetes, is characterized by damage to the retinal microvasculature, which can eventually lead to impaired vision and blindness [1]. In addition to producing vascular dysfunction in the retina, diabetes also damages the neurons [2]. The purpose of investigating transcriptional gene expression was to concurrently measure changes to the neurons, glia, and vasculature as diabetes progresses in the rat retina. These genes included those related to glutamate neurotransmission and transport. The expression of vascular endothelial growth factor (VEGF), erythropoietin (EPO), and insulin-like growth factor-1 (IGF-1) were also measured since they have neuroprotective properties in addition to their effects on the retinal vasculature.

Glutamate is the predominant excitatory neurotransmitter in the retina [3]. This study focused on ionotropic glutamate receptors, which are divided into three classes based on their affinity for the glutamatergic agonists N-methyl-D-aspartate (NMDA), α-amino-3-hydroxy-5-methyl-4-isoxazole propionate (AMPA), and kainate [3]. In previous work, diabetes was found to alter the expression of selected glutamate receptor subunits in the retina of Wistar rats with up to 4 months of diabetes [4,5] and in diabetic patients without signs of retinopathy [6], but an overall pattern of changes was not apparent. The NMDA receptor antagonist memantine was shown to reduce retinal vascular and neuronal changes in a rat model of diabetes [7]; therefore, understanding ionotropic glutamate receptor dysfunction in diabetes may have therapeutic importance. Glutamate transporters are also key constituents in glutamatergic neurotransmission because they regulate the extracellular concentration of glutamate. The glutamate transporter SLC1A3 (also known as GLAST) takes up extracellular glutamate into Müller cells [8]. Another type, the vesicular glutamate transporters (VGLUTs), mediates glutamate uptake into the synaptic vesicles of excitatory neurons [9]. Previous work showed that diabetes impairs glutamate metabolism and transport in the retina [10-13].

To provide further information on retinal and glial cells, the transcript levels of the neural- and glial-related genes γ-synuclein (SNCG), glial fibrillary acidic protein (GFAP), and adenosine A1 receptor (ADORA1) were measured. SNCG is expressed in retinal ganglion cells [14]. It is implicated in the pathogenesis of breast tumors and Alzheimer disease, but the normal physiologic function of SNCG is unknown [15]. GFAP is an intermediate filament protein expressed in glial cells. Increased protein levels of GFAP, which indicates glial reactivity, were found in one study of early diabetic retinopathy [12]. ADORA1 is expressed on retinal ganglion cells and blood vessels [16].

VEGFA and EPO have neuroprotective and angiogenic properties. In addition to being found in retinal blood vessels, VEGF expression has been found in several retinal layers, including the inner nuclear layer (INL), outer nuclear layer (ONL), and ganglion cell layer (GCL), and within the cytoplasm of retinal ganglion cells and glial cells [17-19]. Similar to VEGFA, EPO has angiogenic properties [20]. It is mainly expressed in the kidney, but the retina also expresses EPO along with the EPO receptor (EPOR) [21,22]. In addition to effects on the vasculature, VEGFA and EPO have neuroprotective effects in the brain and retina [23-26]. Similar to VEGF and EPO, the IGF-1 system is involved in angiogenesis and neuroprotection [27]. IGF-1 is normally bound to one of six binding proteins (IGFBP1–6), which prolongs its half-life in the circulation. The IGFBPs also function independently of IGF-1 [28], although their roles have not been fully elucidated. A study of patients with proliferative diabetic retinopathy showed that they had significantly increased IGF-1 and IGFBP2 protein levels in the vitreous [29].

This study measured transcriptional gene expression in the pigmented Long-Evans rat retina at 4 and 12 weeks of diabetes. The results showed concurrent changes in the expression of genes related to glutamate neurotransmission, glutamate transport, VEGF, EPO, and IGFBPs.

## Methods

### Induction of diabetes

These experiments were approved by the Northwestern University IACUC and conformed to the NIH Guide for the Care and Use of Laboratory Animals. Pigmented Long-Evans rats between 50 and 57 days old (Harlan Laboratories, Madison, WI) were maintained on a 12 h:12 h light-dark cycle, and had access to standard rat chow and water ad libitum. The rats were assigned to four groups: 4-week control rats, 4-week diabetic rats, 12-week control rats, and 12-week diabetic rats. Each group comprised of six rats. Diabetes was induced with a single intraperitoneal (IP) injection of streptozotocin (STZ; Axxora, San Diego, CA; 65 mg STZ/kg rat, 6.5 mg/ml) in 0.05 M sodium citrate buffer (pH 5). Rats with blood glucose levels greater than 300 mg/dl 2 days after induction were deemed diabetic. Three rats each from the 4-week diabetic and 12-week diabetic groups had blood glucose levels below 300 mg/dl and were reinjected with STZ. None of the rats were treated with insulin. Age-matched control rats received a single IP injection of an equivalent volume of sodium citrate buffer (0.01 ml/g rat). Blood glucose levels were measured from the tail vein 2 days after the injections and weekly thereafter using a Bayer CONTOUR Meter (Bayer HealthCare, Mishawaka, IN). The meter read “HI” if blood glucose exceeded 600 mg/dl. Those readings were set to 600 mg/dl for averaging. Readings were usually taken in the morning under non-fasting conditions. Rats were also weighed every week.

### Sample collection and preparation

After 4 and 12 weeks of diabetes, the rats were anesthetized with 5% isoflurane and decapitated. Each retina was immediately dissected from the eye as previously described by Winkler [30]. The retina was then frozen on dry ice and stored at −80 °C.

### Quantitative real-time reverse-transcription polymerase chain reaction

Total RNA was extracted from the right retinas using RNeasy Lipid Tissue Mini Kit (Qiagen, Valencia, CA). The cDNA was synthesized by reverse transcription of 1 µg RNA primed with oligo(dT) and random 9-mers. The primers were designed using PerlPrimer [31] as previously described [32]. The forward and reverse primers were limited to 18–20 base pairs (bp) in length. The generated amplicon varied from 69 to 110 bp. The primer sequences and PCR conditions for each gene are given in Appendix 1. The cDNA synthesized from the samples was used as a substrate for quantifying messenger RNA (mRNA) expression levels by quantitative RT–PCR in the presence of SYBR Green (Stratagene, La Jolla, CA). The amount of mRNA of each gene was normalized to acidic ribosomal phosphoprotein (P0) mRNA for each rat [33]. Then, data from the six rats in each group were averaged. Graphs in the Results section show the normalized mRNA levels and relative mRNA scaled to 4-week control rats.

### VEGF protein measurements

Protein was extracted from the left retinas by homogenization in lysis buffer (10 mM Tris pH 7.4, 1.0 mM Na_3_VO_4_, and 1% sodium dodecyl sulfate) at 95 °C. The lysates were incubated at 95 °C for 5 min. The samples were then centrifuged and the supernatant collected. Protein samples were stored at −80 °C until analyzed. Sodium dodecyl sulfate was removed using Pierce Detergent Removal Spin Columns (Pierce Biotechnology, Rockford, IL). Total protein concentration was quantified using the Pierce bicinchoninic acid (BCA) Protein Assay Kit (Pierce Biotechnology). The Quantikine Rat VEGF Immunoassay (R&D Systems, Minneapolis, MN) was used to quantify the concentration of VEGF protein in each retina. The antibody in the immunoassay recognized the VEGFA 120 and 164 isoforms. VEGF protein concentration was then normalized to the total protein concentration for each rat.

### Statistics

All values are reported as mean ± standard error of the mean (SEM) unless otherwise stated. A data point was considered an outlier if it was greater than two standard deviations from the mean of the group. Data sets with outliers were GRIN2D, GRIA2, VGLUT2, VGLUT3, insulin-like growth factor binding protein 2 (IGFBP2), and IGFBP3. These data sets were Winsorized at the fifth percentile [34,35] to minimize the effects of the outlier. The first step in the Winsorization process was to first sort all 24 measurements in a data set from lowest to highest. Then, the lowest and highest values were replaced with the next value in the data set. Thus, the mRNA levels for the five genes listed above are reported as the Winsorized mean and SEM. The data for IGFBP2 were averaged from two separate qRT-PCR runs. Statistical significance was determined using a two-factorial analysis of variance (ANOVA) with two levels in each factor (2×2 ANOVA) and was defined as p<0.05. The factors for the ANOVA were time point (levels: 4 weeks and 12 weeks) and treatment (levels: control and diabetic). Fisher’s protected least significant difference was used for post-hoc analysis. StatView (SAS Institute, Cary, NC) was used to perform the statistical analyses.

## Results

### Streptozotocin-induced diabetes

**Figure 1:**
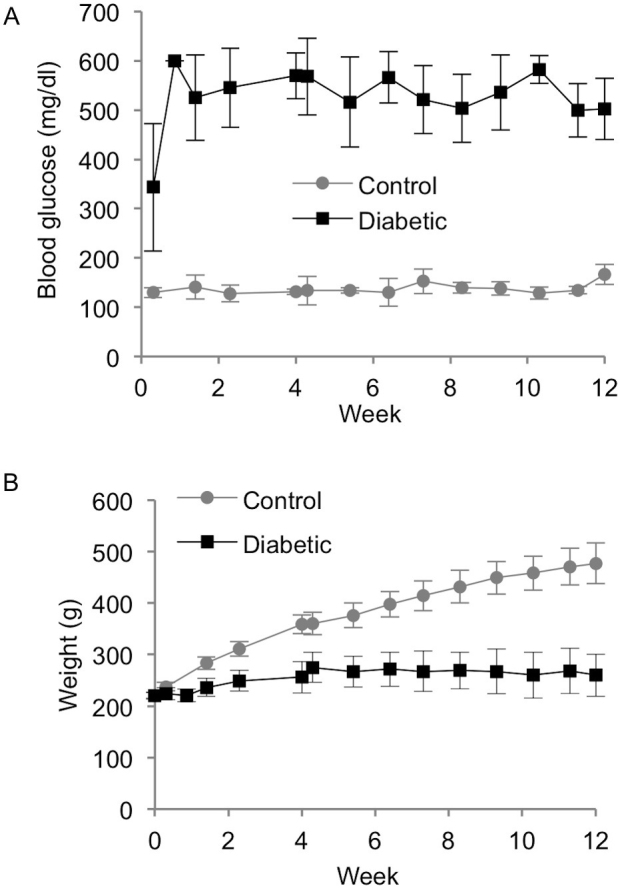
Blood glucose and weight. Weekly measurements of (**A**) blood glucose and (**B**) weight for control and diabetic rats combined for 4- and 12-week time points (mean±SD).

All the STZ-treated rats exhibited characteristics of diabetes. The rats’ blood glucose levels were over 300 mg/dl and remained consistently hyperglycemic until the animals were euthanized (Figure 1A). Several rats lost weight after STZ treatment, and all the diabetic rats gained weight slower than the age-matched control rats (Figure 1B). They also showed symptoms of polyuria. The age-matched control rats had normal glucose levels, consistently gained weight until euthanized, and showed no signs of polyuria.

### Transcriptomic analyses

The significant changes in mRNA expression found from post-hoc tests following ANOVA are discussed below. The complete results of the ANOVA are summarized in Appendix 2.

### NMDA receptor subunits

**Figure 2:**
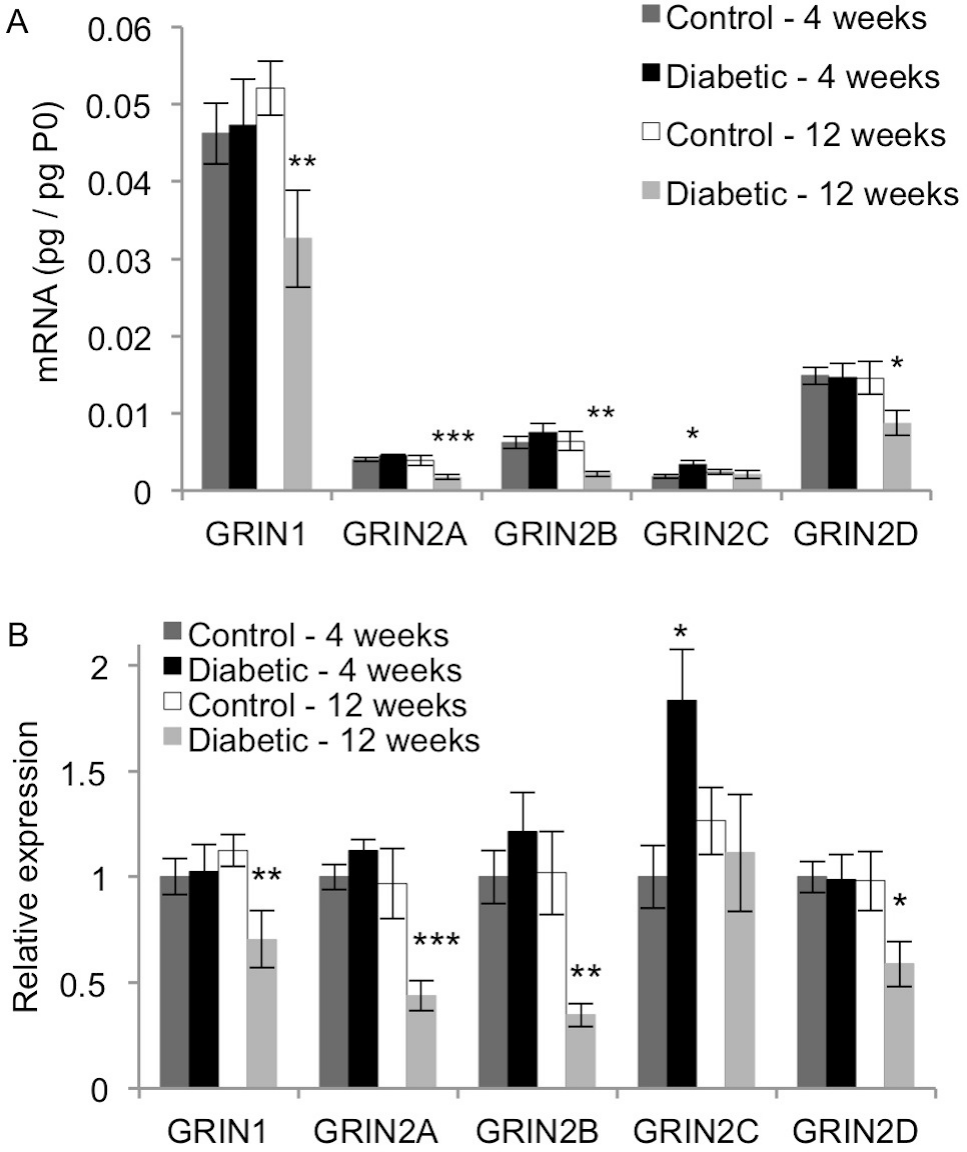
Effect of diabetes on expression of NMDA receptor subunits. qRT-PCR analysis was performed on cDNA isolated from control and STZ-induced diabetic rat retina after 4 and 12 weeks. Expression of each gene was normalized to acidic ribosomal phosphoprotein (P0) for each rat (**A**), and then scaled to the 4-week control rats for each gene (**B**; mean ± SEM). Compared to the age-matched control rats, the 12-week diabetic rats had significantly reduced transcript levels of GRIN1, GRIN2A, GRIN2B, and GRIN2D (*, p<0.05; **, p<0.01; *** p<0.005). Transcript levels of GRIN2C were significantly increased in the 4-week diabetic rats compared to the age-matched control rats (*, p<0.05).

All the ionotropic glutamate receptors are tetrameric proteins that form cation channels. The NMDA receptor is a heterotetramer formed by two conserved NR1 subunits encoded by the gene GRIN1 and two NR2 subunits encoded by the genes GRIN2A–D [36]. GRIN1 is more abundantly expressed in the retina than the other subunits (Figure 2A). Its expression levels in the 12-week diabetic rats were significantly lower than in the 12-week control rats and the 4-week diabetic rats (p<0.05). The 12-week diabetic rats also had lower GRIN1 mRNA levels than the 4-week control rats, but the difference did not reach significance (p=0.0610). For the genes GRIN2A, GRIN2B, and GRIN2D, the transcript levels were significantly decreased in the 12-week diabetic rats compared to each of the three other groups (p<0.002, p<0.01, and p<0.03, respectively). The mRNA expression pattern for GRIN2C differed from that of the other NMDA receptor subunits (Figure 2B). GRIN2C was significantly increased in the 4-week diabetic rats compared to the age-matched control rats (p<0.02).

### AMPA receptors

**Figure 3:**
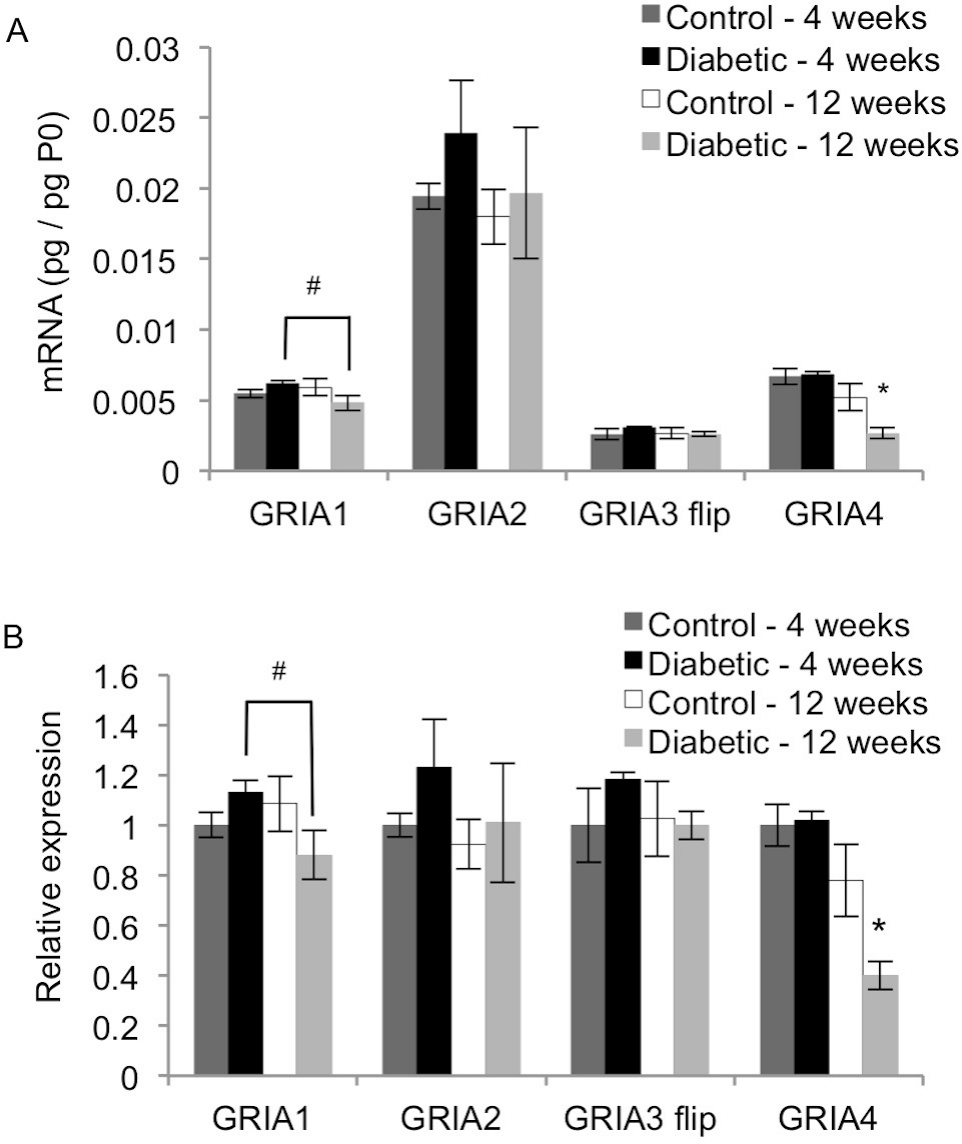
Effect of diabetes on mRNA expression of AMPA receptor subunits. qRT-PCR analysis was performed on cDNA isolated from control and STZ-induced diabetic rat retinas after 4 and 12 weeks. Expression of each gene was normalized to acidic ribosomal phosphoprotein (P0) for each rat (**A**), and then scaled to the 4-week control rats for each gene (**B**; mean ± SEM). The 12-week diabetic rats had significantly lower GRIA1 mRNA than the 4-week diabetic rats (#; p<0.05). The 12-week diabetic rats also had significantly reduced mRNA levels of GRIA4 compared to the age-matched control rats (**, p<0.01). Diabetes did not affect the mRNA levels of GRIA2 and GRIA3 flip at 4 or 12 weeks.

Similar to the NMDA receptors, the AMPA receptors are heterotetramers. Each AMPA receptor is composed of two conserved subunits of GluR2, which is encoded by GRIA2 and is the most abundantly expressed subunit in the retina (Figure 3A). The other two subunits are GluR1, GluR3, or GluR4, which are encoded by GRIA1, GRIA3, and GRIA4, respectively. The AMPA receptor subunits each have two isoforms, flip and flop, which result from alternative splicing of the mRNA transcript [37]. The retina predominantly expresses the flop isoforms of GRIA1, GRIA2, and GRIA4, and the flip and flop isoforms of GRIA3 [38]. The primers for GRIA1, GRIA2, and GRIA4 were not specific for a particular isoform, while the flip isoform of GRIA3 was analyzed. Figure 3B shows the expression patterns of the AMPA receptors scaled to the 4-week control rats for each gene. The 12-week diabetic rats had significantly lower GRIA1 transcript levels than the 4-week diabetic rats (p<0.05). The 12-week diabetic rats also had significantly lower expression of GRIA4 mRNA than each of the three other groups (p<0.01). In contrast, diabetes did not alter the expression of GRIA2 and GRIA3 flip at either 4 or 12 weeks (p>0.02).

### Kainate receptors

**Figure 4:**
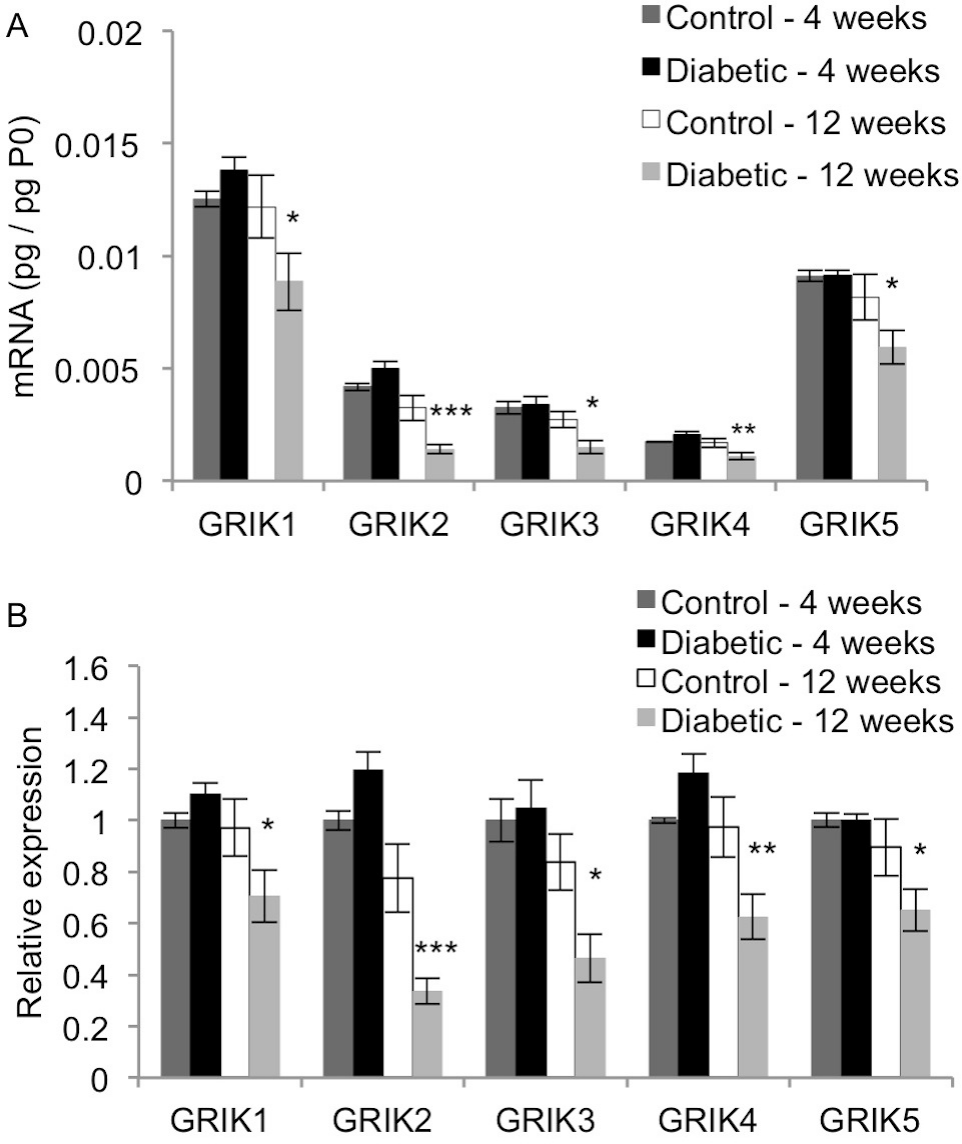
Effect of diabetes on expression of the kainate receptor subunits. qRT-PCR analysis was performed on cDNA isolated from control and STZ-induced diabetic rat retinas after 4 and 12 weeks. Expression of each gene was normalized to acidic ribosomal phosphoprotein (P0) for each rat (**A**), and then scaled to the 4-week control rats for each gene (**B**; mean ± SEM). Compared to the age-matched control rats, the 12-week diabetic rats had significantly reduced mRNA levels of all the kainate receptor subunits (*, p<0.05; **, p<0.01; ***, p<0.005).

The genes GRIK1, GRIK2, GRIK3, GRIK4, and GRIK5 encode for the protein subunits GluR5, GluR6, GluR7, KA1, and KA2, respectively. GluR5, GluR6, and GluR7 can form homomers and heteromers, whereas KA1 and KA2 must complex with GluR5, GluR6, or GluR7 to form a functional receptor [39]. Figure 4A shows the abundance of transcript for each kainate receptor subunit. All five kainate receptor subunits showed similar mRNA expression patterns (Figure 4B) where the 12-week diabetic rats had significantly lower mRNA levels than each of the three other groups (p<0.03).

### Glutamate transporters

**Figure 5:**
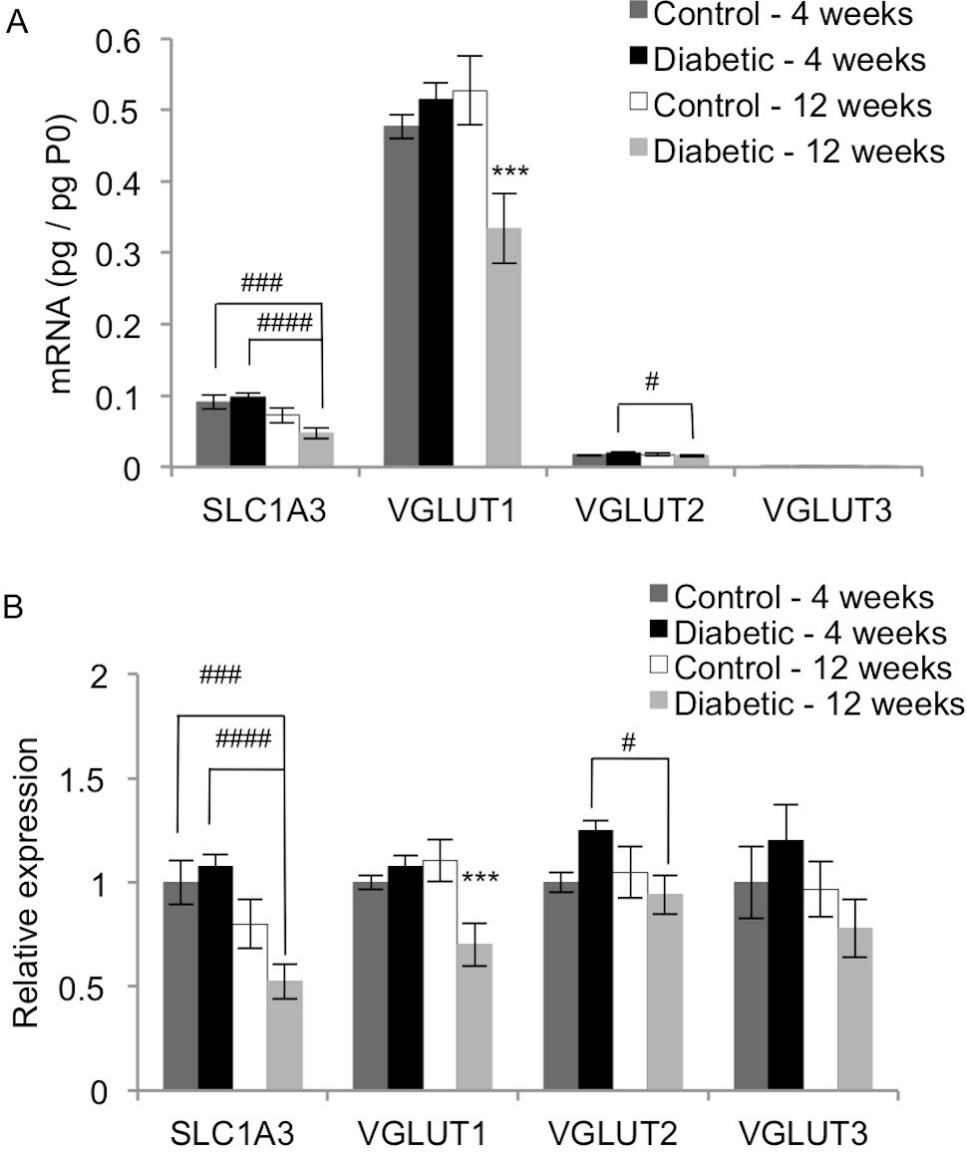
Effect of diabetes on expression of the glutamate transporters SLC1A3, VGLUT1, VGLUT2, and VGLUT3. qRT-PCR analysis was performed on cDNA isolated from control and STZ-induced diabetic rat retinas after 4 and 12 weeks. Expression of each gene was normalized to acidic ribosomal phosphoprotein (P0) for each rat (**A**), and then scaled to the 4-week control rats for each gene (**B**; mean ± SEM). The SLC1A3 mRNA levels in the 12-week diabetic rats were significantly lower than those of the 4-week control rats and 4-week diabetic rats (###, p<0.005; ####, p<0.001). The 12-week diabetic rats also had lower SLC1A3 mRNA levels than the 12-week control rats, but the difference was not quite significant (p=0.0522). The 12-week diabetic rats had significantly lower VGLUT1 mRNA levels than the age-matched control rats (***, p<0.005). VGLUT2 mRNA was significantly higher in the 4-week diabetic rats compared to the 12-week diabetic rats (#, p<0.05). VGLUT2 mRNA levels in the 4-week diabetic rats increased 1.25 fold over the 4-week control rats, but the difference was not quite significant (p=0.0503).

The mRNA expression levels of four glutamate transporters were measured: SLC1A3, VGLUT1, VGLUT2, and VGLUT3 (Figure 5). SLC1A3 is expressed on Müller cells and is responsible for uptake of glutamate for reprocessing [8]. SLC1A3 mRNA levels in 12-week diabetic rats were significantly lower than those of the 4-week control rats and the 4-week diabetic rats (p<0.005 and p<0.001, respectively). The 12-week diabetic rats also had lower SLC1A3 mRNA levels than the 12-week control rats, with the difference approaching significance (p=0.0522). The VGLUTs mediate glutamate uptake into synaptic vesicles. VGLUT1 mRNA was more abundantly expressed than the two other vesicular transporters (Figure 5A), and its expression was significantly lower in 12-week diabetic rats than each of the three other groups (p<0.05). VGLUT2 expression was significantly decreased in the 12-week diabetic rats compared to the 4-week diabetic rats (p<0.02). VGLUT2 mRNA levels in 4-week diabetic rats trended toward a 1.25-fold increase over the 4-week control rats (p=0.0503). In contrast to its effect on the other glutamate transporters, diabetes had no effect on the mRNA expression of VGLUT3 (p>0.15).

### SNCG, GFAP, and ADORA1

**Figure 6:**
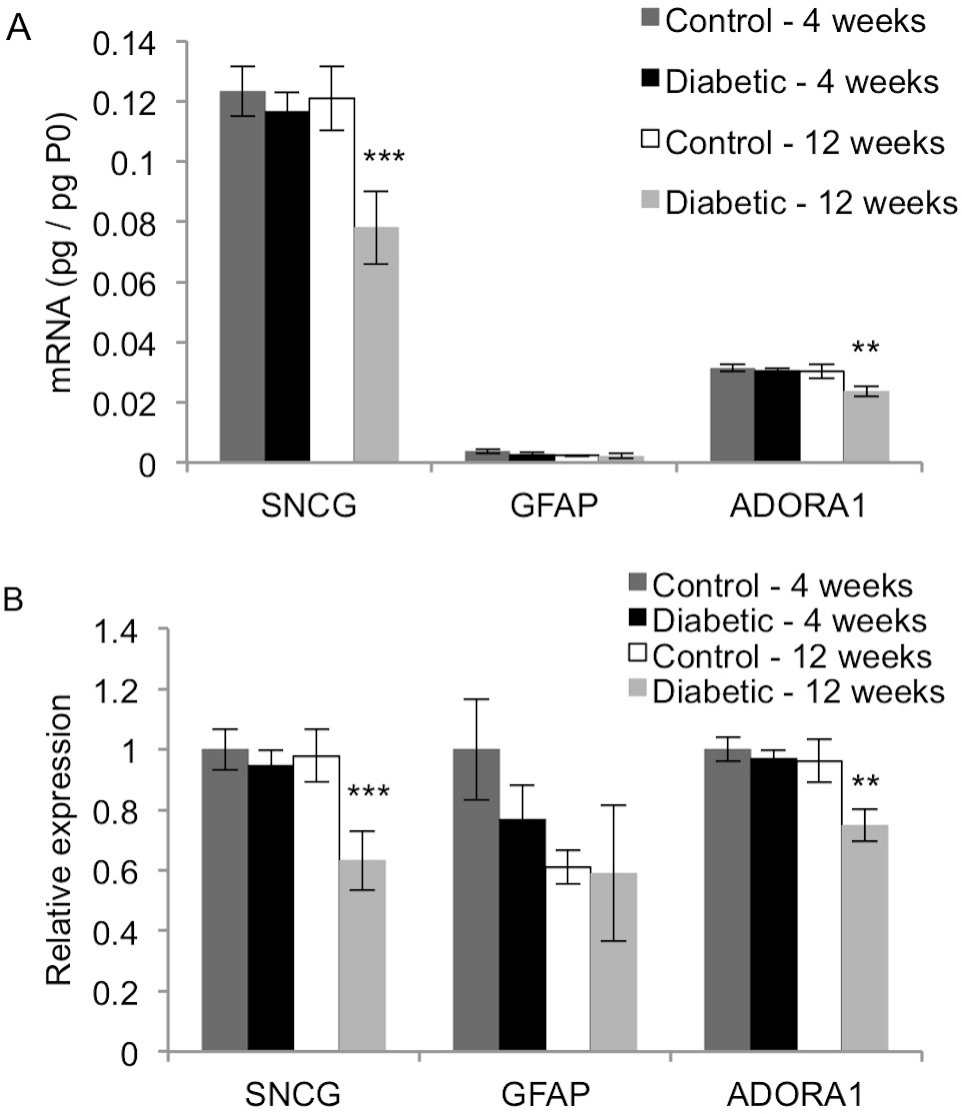
Effect of diabetes on expression of SNCG, GFAP, and ADORA1. qRT-PCR analysis was performed on cDNA isolated from control and STZ-induced diabetic rat retinas after 4 and 12 weeks. Expression of each gene was normalized to acidic ribosomal phosphoprotein (P0) for each rat (**A**), and then scaled to the 4-week control rats for each gene (**B**; mean ± SEM). The 12-week diabetic rats had significantly lower SNCG and ADORA1 mRNA levels than the age-matched control rats (**, p<0.01; ***, p<0.005). Diabetes did not affect the mRNA levels of GFAP at 4 or 12 weeks.

The mRNA expression patterns of other neural- and glial-related genes not directly connected to glutamate signaling were also studied. Figure 6A shows the relative transcript levels of each gene in the retina, and Figure 6B shows their expression patterns. The 12-week diabetic rats had significantly lower levels of SNCG and ADORA1 mRNA than each of the three other groups (p<0.01). In contrast, diabetes did not affect GFAP expression at either 4 or 12 weeks of diabetes (p>0.05).

### VEGF, EPO, and their receptors

**Figure 7:**
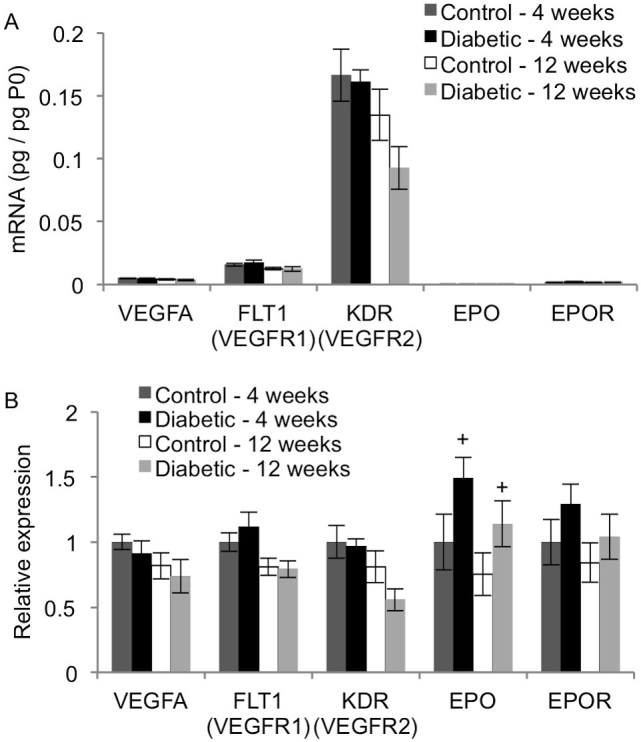
Effect of diabetes on expression of VEGF and VEGF–associated genes. qRT-PCR analysis was performed on cDNA isolated from control and STZ-induced diabetic rat retinas after 4 and 12 weeks. Expression of each gene was normalized to acidic ribosomal phosphoprotein (P0) for each rat (**A**), and then scaled to the 4-week control rats for each gene (**B**; mean ± SEM). The retina expresses erythropoietin (EPO) at low levels, yet diabetes significantly increased EPO transcript levels (significant main effect for treatment factor; +, p<0.05). The transcriptional expression of VEGFA, the vascular endothelial growth factor (VEGF) receptors FLT1 and KDR, and erythropoietin receptor (EPOR) did not change with diabetes.

The relative transcript levels for VEGF, EPO, and their respective receptors are shown in Figure 7A. Figure 7B shows the expression patterns. Diabetes did not affect VEGFA mRNA levels (p>0.05). The VEGF receptors FLT1 and KDR significantly decreased after 12 weeks in the control and diabetic rats (p<0.02), which is most likely due to age effects, not diabetes. Diabetes significantly increased EPO mRNA levels 1.49-fold after 4 weeks and 1.51-fold after 12 weeks (p<0.05). Unlike its ligand, EPO receptor (EPOR) expression did not change with diabetes (p>0.15).

### IGF-1 receptor and binding proteins

**Figure 8:**
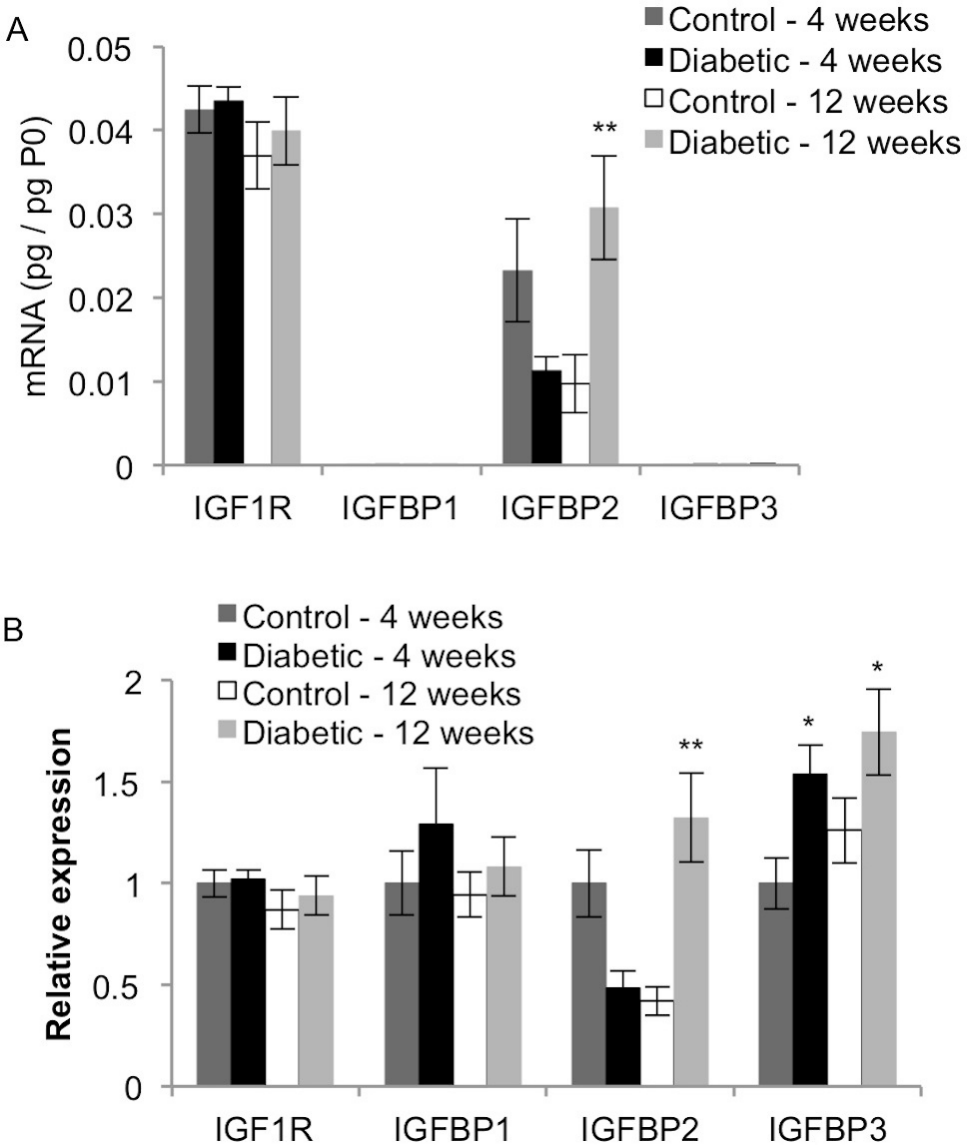
Effect of diabetes on mRNA expression of IGF-1 associated genes. qRT-PCR analysis was performed on cDNA isolated from control and STZ-induced diabetic rat retinas after 4 and 12 weeks. Expression of each gene was normalized to acidic ribosomal phosphoprotein (P0) for each rat (**A**), and then scaled to the 4-week control rats for each gene (**B**; mean ± SEM). The 12-week diabetic rats had significantly higher IGFBP2 transcript levels than the age-matched control rats (**, p<0.01). The 4-week diabetic rats had lower IGFBP2 mRNA levels than the 4-week control rats, but the difference did not reach significance (p=0.0532). Although expression of IGFBP3 in the retina was low, the IGFBP3 transcript levels for the 4-week and 12-week diabetic rats were significantly increased over those of the age-matched control rats (*, p<0.05). Diabetes did not affect the mRNA expression levels of IGF1R or IGFBP1 at 4 or 12 weeks.

IGFBP2 transcript levels were much greater than the levels of IGFBP1 and IGFBP3 (Figure 8A). IGFBP2 mRNA levels were significantly higher in the 12-week diabetic rats than in the age-matched control rats and the 4-week diabetic rats (p<0.02). The 4-week diabetic rats trended toward lower IGFBP2 mRNA levels than age-matched control rats (p=0.0532). Although its expression was low in the retina, IGFBP3 mRNA levels in the 4-week and 12-week diabetic rats were significantly increased over their age-matched control rats (p<0.05). The effect of diabetes on mRNA expression at each time point varied among IGF1R, IGFBP1, IGFBP2, and IGFBP3 (Figure 8B). Diabetes did not alter the mRNA expression of IGF1R or IGFBP1 (p>0.15).

### VEGF protein levels

**Figure 9:**
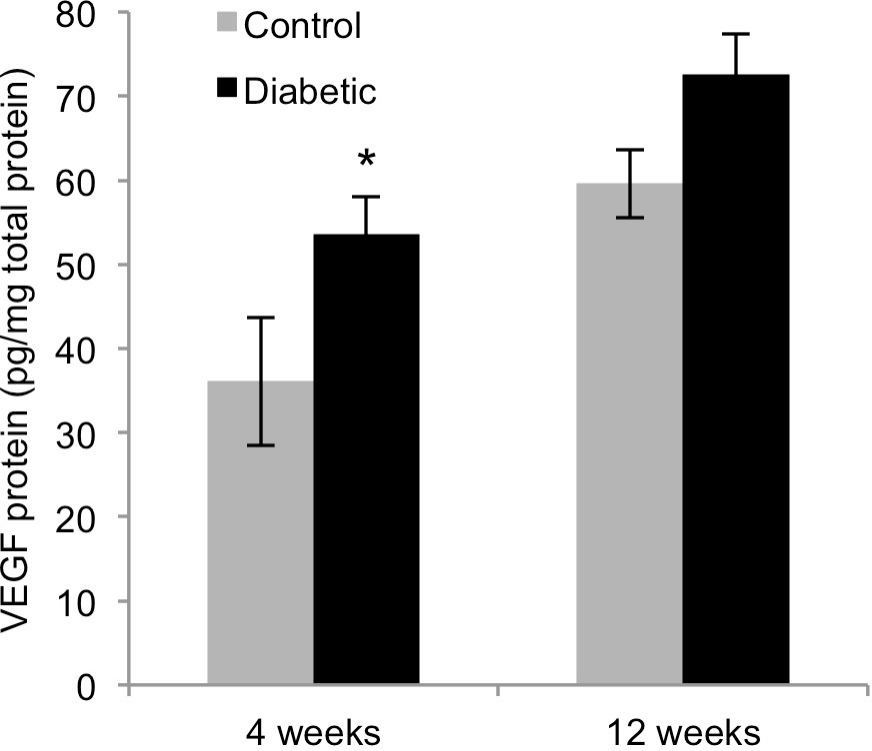
Effect of diabetes on VEGF protein levels. Total protein was extracted from one retina of each rat. VEGF protein levels were measured with enzyme-linked immunosorbent assay (ELISA) and normalized to total protein for diabetic (black bars) and age-matched control (gray bars) rats at 4 and 12 weeks (mean ± SEM). The asterisk (*) indicates significantly different from age-matched control rats, p<0.05).

The diabetic rats had elevated VEGF protein levels compared to the age-matched control rats. Figure 9 shows VEGF protein levels normalized to total protein for each group. The 4-week diabetic rats had 1.5 times the VEGF protein of the age-matched control rats (53.5±4.6 pg VEGF/mg total protein versus 36.1±7.6 pg VEGF/mg total protein, p<0.04). The 12-week diabetic rats also had higher VEGF protein levels at 1.2 times the levels of the age-matched control rats (72.5±4.9 pg VEGF/mg total protein versus 59.6±4.1 pg VEGF/mg total protein), but this difference was not significant (p=0.1025). Total protein levels were not significantly different between the groups (p>0.1).

## Discussion

Diabetic retinopathy is clinically defined as injury to the retinal microvasculature. In addition to vascular changes, patients with diabetes demonstrate retinal functional changes, which can appear early in non-proliferative diabetic retinopathy (NPDR) before signs of microvascular injury. Thus, dysfunction in the diabetic retina encompasses vascular and neural changes [2]. This study evaluated genes related to glutamate neurotransmission and transport, and genes that have protective effects on the retinal vasculature and neurons (Table 1). Several studies have shown that diabetes increased apoptosis and ganglion cell loss in the rat retina. STZ-induced diabetes significantly increased TUNEL–positive cells in Sprague-Dawley rat retinas after 1, 3, 6, and 12 months of diabetes [40]. Another group found significantly lower retinal ganglion cell counts after 4 weeks of diabetes in Brown Norway rats [7]. Likewise, diabetic patients exhibit structural changes to the inner retina early in diabetes. Patients with non-proliferative diabetic retinopathy had a significantly thinner nerve fiber layer (NFL) than non-diabetic control rats [41-44]. In patients with minimal NPDR, the GCL and the NFL were significantly thinner than in non-diabetic control rats, while the outer retina was unaffected [45-49]. Thinning of the GCL and the NFL suggests that ganglion cell injury or death occurs early in diabetes.

**Table 1:** Summary of gene expression changes.

**Decreased expression at 12 weeks of diabetes**		**Interaction between time point and diabetes: Increase at 4 weeks followed by decrease at 12 weeks**
NMDA receptor subunits	GRIN1	GRIA1
	GRIN2A	VGLUT2
	GRIN2B	LDHB
	GRIN2D	GAPDH
AMPA receptor subunit	GRIA4	**Increased expression at 4 weeks of diabetes**
Kainate receptor subunits	GRIK1	GRIN2C
	GRIK2	**Increased expression at 12 weeks of diabetes**
	GRIK3	IGFBP2
	GRIK4	**Increased with diabetes**
	GRIK5	EPO
Glutamate transporters	SLC1A3	IGFBP3
	VGLUT1
Neural-related	SNCG
	ADORA1

The results of this study combined with previous work suggest that the loss of ganglion cells in diabetes may be caused by glutamate excitotoxicity [50-52]. Most glutamate receptor subtypes have been implicated in excitotoxicity by allowing excessive influx of Ca^2+^ into neurons [50]. Elevated intracellular calcium levels can trigger various downstream effects, including cell death. Although the exact mechanisms leading from excess glutamate to cell death are not fully understood, Ca^2+^ influx through NMDA receptors is a key contributor [52]. NMDA receptors are the primary mediators because they are directly coupled to Ca^2+^ signaling pathways that lead to cell death [50,52]. Thus, the pathway of Ca^2+^ influx through NMDA receptors is pathologically more detrimental than the concentration of intracellular Ca^2+^.

### Glutamate transporters

Diabetes was previously found to impair glutamate transport and glutamate recycling in Müller cells [11,12]. Müller cells maintain a low extracellular concentration of glutamate by taking it up via the transporter SLC1A3, also known as GLAST [8]. The activity of SLC1A3 in Müller cells isolated from Long-Evans rats was reduced after 4 weeks and decreased further after 13 weeks [11]. Consistent with those results, this study found that SLC1A3 mRNA levels were significantly reduced after 12 weeks of diabetes. The changes in SLC1A3 expression are most likely specific to that gene and do not reflect a general loss of Müller cells since the GFAP mRNA levels were not significantly altered. Within the Müller cells, glutamine synthetase converts glutamate to the less neuroactive glutamine, which is then taken up by neurons and converted to glutamate. The content and activity of glutamine synthetase in the retina decreased after 2, 3, and 6 months of diabetes in Sprague-Dawley rats [13]. These Müller cell dysfunctions in diabetes may lead to accumulation of glutamate in the extracellular space of the retina and contribute to glutamate excitotoxicity [10,12,13].

In addition to SLC1A3, the expression of VGLUT1 and VGLUT2 transcripts was also altered by STZ-induced diabetes. The main function of the VGLUTs is to load glutamate from the cytoplasm into synaptic vesicles [9]. In the rat retina, VGLUT1 is expressed in photoreceptor and bipolar cell terminals [53]. In this study, VGLUT1 expression was significantly decreased after 12 weeks of diabetes. In contrast, VGLUT2 mRNA was upregulated after 4 weeks of diabetes, but the increase was not sustained after 12 weeks. VGLUT2 is expressed on horizontal and ganglion cells in the rat retina [53]. VGLUT3 is expressed in non-glutamatergic amacrine cells in the rat retina [53], and its mRNA expression was not affected by diabetes in this study. Diabetes decreased VGLUT1 and VGLUT2 protein levels in retinal synaptosomes after 2 weeks but not after 8 weeks in Wistar rats [54]. Diabetes affects the expression of VGLUT1 and 2, but more studies are needed to determine the pathological consequences.

### Ionotropic glutamate receptors

Diabetes also altered the expression of the NMDA receptor subunit transcripts. With the exception of GRIN2C, the NMDA receptor subunits showed significantly reduced mRNA expression after 12 weeks of diabetes. However, in contrast to the results of this study, another study using Wistar rats found that GRIN1 mRNA expression was increased after 1 and 4 weeks of diabetes and did not change after 12 weeks, and GRIN2C mRNA levels did not change at any time point [4]. Retinal ganglion cells express the NMDA receptor subunits GRIN1 and GRIN2A-D, but the combination of NMDA receptor subunit expression can depend on the individual cell [38]. GRIN1, GRIN2A, GRIN2B, and GRIN2D are much more likely to be expressed on ganglion cells than GRIN2C. Conversely, amacrine cells also express GRIN1 and GRIN2A–D but NMDA receptors are not found in all amacrine cells [38]. It is unclear why GRIN2C had a different expression pattern than the other NMDA receptor subunits. GRIN2C mRNA increased after 4 weeks of diabetes, but qRT-PCR of the entire retina cannot distinguish whether ganglion cells or amacrine cells were responsible. The depressed expression of GRIN1, GRIN2A, GRIN2B, and GRIN2D mRNA could indicate downregulation of NMDA receptors but, taken with other evidence, strongly indicates ganglion cell and possibly amacrine cell loss after 12 weeks. Ganglion cells may be more susceptible to glutamate excitotoxicity than other neurons because they are the primary cell type expressing NMDA receptors.

Ganglion cells also express AMPA and kainate receptors, as do other retinal neurons. In situ hybridization studies showed strong labeling for GRIK1, GRIK2, GRIK3, and GRIK5 mRNA in ganglion cells [36]. In the present study, all the kainate receptor subunits were downregulated after 12 weeks of diabetes.

Most of the AMPA receptor subunits exhibited different mRNA expression patterns than the kainate and NMDA receptor subunits, which may be due to differences on which retinal cell types the subunits are expressed. In situ hybridization showed that GRIA2 and GRIA3 were expressed in the cells of the INL, the ONL, and some ganglion cells [55]. In this study, diabetes did not change the expression of GRIA2 and GRIA3. In agreement with these results, the GRIA2 and GRIA3 mRNA levels did not change in the Long-Evans rat retina assessed with in situ hybridization after 2 and 6 weeks of diabetes [55], nor did they change in the Wistar rat retina after 1, 4, or 12 weeks of diabetes as measured with qRT-PCR [4]. In addition to ganglion cells, photoreceptors, bipolar cells, and amacrine cells express GRIA1 and GRIA3. Diabetes did not change GRIA2 and GRIA3 mRNA expression possibly because photoreceptors, bipolar cells, and amacrine cells were less likely to be affected by glutamate excitotoxicity than ganglion cells. GRIA1 is expressed predominantly by amacrine cells and bipolar cells, and to a lesser extent by ganglion cells [38]. The mRNA expression of GRIA1was biphasic with an increase after 4 weeks and a decrease after 12 weeks. However, Wistar rats showed no change in GRIA1 transcript levels after 1, 4, and 12 weeks of diabetes [4]. GRIA4 is almost exclusively expressed by ganglion cells [38]. Its mRNA expression levels were significantly decreased after 12 weeks of diabetes, supporting the conclusion that ganglion cells were lost at that time point in Long-Evans rats.

Although there could be selective downregulation of GRIA4 and specific NMDA and kainate receptor subunits with no loss of ganglion cells, it is more likely that the ganglion cells expressing these receptors were lost by 12 weeks of diabetes. The evidence that glutamate uptake is reduced implicates glutamate excitotoxicity in this process. With continued elevation of glutamate, ganglion cell loss would be expected to continue past the time points investigated here. Other evidence obtained in this study supports the conclusion that ganglion cells were lost in the 12-week diabetic rat retina. SNCG was used as a marker for ganglion cells [14], and its mRNA levels decreased significantly in the 12-week diabetic rats compared to each of the three other groups. ADORA1 mRNA levels also decreased significantly in the 12-week diabetic rats. It is expressed in the ganglion cell layer [16], and interaction with adenosine reduces glutamate-induced calcium influx into the ganglion cells [56]. As noted in the introduction, other work also supports the conclusion that ganglion cells are lost as diabetes progresses in rodent models and humans. The loss of ganglion cells could partially account for the decreased visual function of diabetic Long-Evans rats [57]. At 8 weeks of diabetes, rats with or without cataracts exhibited similar losses in contrast sensitivity and acuity.

### The VEGF, EPO, and IGF-1 system

In this study, VEGF protein levels significantly increased after 4 weeks but not 12 weeks of diabetes, which is consistent with other studies. The VEGF protein levels were higher in the diabetic rats than the control Sprague-Dawley rats after 2, 4, and 6 weeks of diabetes but not after 12 weeks [58]. Another study found that VEGF protein levels were increased after 4 weeks of diabetes in Sprague-Dawley and Long-Evans rats and significantly in Brown Norway rats, yet no change was found in any strain after 12 weeks [59]. The early increase in VEGF protein levels was not accompanied by an increase in mRNA expression in this study or the study by Schrufer et al. [58], while Brucklacher et al. found VEGF mRNA levels decreased in diabetic rats at 4 and 12 weeks [60]. These results suggest that post-transcriptional mechanisms or translational regulation acts to alter VEGF protein levels, or that qRT-PCR of samples from the whole retina is not sensitive enough to detect VEGF mRNA changes occurring in specific cell types. In addition, the results show that elevated VEGF protein levels are not persistent in the retina early in diabetes and can vary as the disease progresses. Diabetes did not alter the mRNA expression of the VEGF receptors FLT1 and KDR.

EPO also has angiogenic properties [20,61,62]. VEGF and EPO are reported to have neuroprotective properties as well [63,64]. In a post-mortem analysis, retinas from diabetic patients without diabetic retinopathy had higher EPO mRNA levels than age-matched controls [21]. In this study, the EPO mRNA levels were elevated in the rat retina at 4 and 12 weeks of diabetes, possibly to protect the neural and vascular cells in the retina.

Diabetes increased the expression of IGFBP2 after 12 weeks and IGFBP3 after 4 and 12 weeks, but did not change the expression of IGF1R or IGFBP1. The interaction between IGF-1 and its receptors regulates VEGF expression and can induce blood–retinal barrier breakdown [65] and retinal neovascularization [66,67]. The effects of the IGF-1 system can be seen in the vasculature and the central nervous system [68,69]. The IGF binding proteins modulate the activity of IGF-1 but also have effects independent of IGF-1 and IGF1R. IGFBP3 has been shown to have anti- and proapoptotic characteristics and to promote and inhibit proliferation in various cell and tissue types (see [70,71] for reviews). These activities are most likely dependent on tissue type and pathological condition. In agreement with this study, Kirwin et al. found IGFBP3 transcript levels significantly increased after 4 weeks and 3 months of diabetes in the Long-Evans rat retina [72]. In the mouse model of retinopathy of prematurity, exogenous IGFBP3 promoted vessel survival during the vaso-obliterative hyperoxic phase and increased vessel regrowth during the relative hypoxic phase independent of IGF-1 [73], and reduced apoptosis in retinal neurons [74]. Even less is known about the functions of IGFBP1 and IGFBP2 in the retina. How IGF binding proteins impact the progression of diabetic retinopathy has yet to be fully evaluated.

### Conclusion

Diabetes caused significant changes in the expression of genes related to glutamate neurotransmission and transport. Evidence suggests diabetes causes dysfunction in glutamate processing resulting in ganglion cell loss. The effect of diabetes on the expression of ionotropic glutamate receptor subunits varies between humans and rats and between rat strains. Nonetheless, diabetes alters the expression of the various ionotropic glutamate receptors, and the changes vary over the duration of diabetes. Mounting evidence indicates that diabetes disrupts glutamate signaling in the retina and affects retinal neurons as well as the retinal vasculature. Alterations in gene expression varied with the duration of diabetes. Most of the genes with elevated mRNA levels after 4 weeks did not have sustained increases after 12 weeks. In addition, more genes had altered expression after 12 weeks, indicating that diabetes leads to more changes in the retina over time. Increased expression of EPO and IGFBP3 and increased VEGF protein levels may be protective responses to damage caused by diabetes, but these responses may not provide sufficient protection. This study shows that diabetes not only injures the retinal vasculature but also affects the neurons in the retina.

## Appendix 1. Primer sequences.

To access the data, click or select the words “Appendix 1.”

## Appendix 2: Results of two-factorial ANOVA (2×2 ANOVA) for each gene.

To access the data, click or select the words “Appendix 2.”
